# Increased CD44s and decreased CD44v6 RNA expression are associated with better survival in myxofibrosarcoma patients: a pilot study

**DOI:** 10.1186/2047-783X-19-6

**Published:** 2014-02-03

**Authors:** Christiane Matuschek, Marcus Lehnhardt, Peter Arne Gerber, Christopher Poremba, Jackson Hamilton, Guido Lammering, Klaus Orth, Wilfried Budach, Hans Bojar, Edwin Bölke, Matthias Peiper

**Affiliations:** 1 Klinik für Allgemein-, Viszeral- und Kinderchirurgie, Universitätsklinikum Düsseldorf, Moorenstraße 5, 40225 Düsseldorf, Germany; 2 Current address: Abteilung für Allgemein-, Viszeral- und Unfallchirurgie, Kliniken Essen-Süd, Propsteistraße 2, 45222 Essen, Germany; 3 Universitätsklinik für Plastische Chirurgie und Schwerbrandverletzte, BG Kliniken Bergmannsheil, Ruhr Universität Bochum, Bürkle-de-la-Camp-Platz 1, 44789 Bochum, Germany; 4 Institut für Pathologie, Universitätsklinikum Düsseldorf, Moorenstraße 5, 40225 Düsseldorf, Germany; 5 Klinik für Strahlentherapie und Radiologische Onkologie, Medical Faculty, Universitätsklinikum Düsseldorf, Moorenstraße 5, 40225 Düsseldorf, Germany; 6 Department of Diagnostic, The University of Texas MD Anderson Cancer Center, Radiology, Houston, Texas, USA; 7 Klinik für Allgemein-, Visceral- und Thoraxchirurgie, Asclepios Harzkliniken, Köslinerstr. 12, Goslar, Germany; 8 Pathologie München-Nord, Ernst-Platz-Str. 2, 80992 München, Germany

**Keywords:** CD44, Malignant fibrous histiocytoma, MFS, Myxofibrosarcoma, Soft tissue sarcoma, Survival, Tumor prognosis

## Abstract

**Background:**

New prognostic markers may be of value in determining survival and informing decisions of adjuvant treatment in the heterogeneous group of soft tissue sarcomas known as malignant fibrous sarcomas (MFS). Increased CD44 expression has been associated with a better outcome in cancers such as bladder tumors and could potentially relate to cell-cell interaction as a marker for potential invasion/metastasis. The aim of this pilot study was to determine if there is a correlation between the expression rate of CD44 in adult patients with MFS and clinical outcomes.

**Methods:**

The clinical outcome of 34 adult MFS patients (19 males and 15 females, average age 62 years, median 63 years, range: 38–88 years) who underwent surgical treatment were evaluated. Twenty-five of these patients had additional adjuvant radiotherapy. Extracted RNA from sarcoma tissues was used to measure the transcripts of CD44s (standard form) and isoform expression.

The pooled data for each variant of CD44 was divided in half at the median expression value into two equally sized groups (low and high). Survival modeling and multivariate analysis were used with these two groups to determine if there were differences in survival times and whether this was independent of known factors such as tumor stage/grade, patient age and resection margin status.

**Results:**

High CD44s and low of CD44v6 expression significantly correlated with an improved outcome (*P* <0.05 and *P* <0.02, respectively) whereas CD44v8 and hCD44 (isoforms) did not. Differences in survival were apparent within 6–12 months of operation with >30% difference in survival between low/high expressions at 5 years. These finding were independent of the other measured MFS survival predictors, though the group was homogenous.

**Conclusions:**

High CD44s and low CD44v6 expression may be an independent predictor of improved survival in MFS patients in this pilot data. This is contrary to other MFS data, which did not account for the CD44 isoforms but is confirmed by data from other cancer types. Further investigation is needed to confirm CD44 isoform expression data as a relevant survival biomarker and whether it could be used to inform clinical decisions such as adjuvant therapy.

## Background

Soft tissue sarcomas (STS) are a clinically and pathologically heterogeneous group of tumors that lead to therapeutic difficulties. The most frequent histological type is malignant fibrous histiocytoma, which is a heterogeneous group in itself, accounting for approximately 30% of STS. The origin of these tumors may come from undifferentiated mesenchymal stem cells from fibroblasts or dual fibroblastic-histiocytic origin [1-11]. Although not presently accepted by all, it has been suggested that the term myxofibrosarcoma or malignant fibrous sarcoma (MFS) should be used for the myxoid variant of malignant fibrous histiocytoma [1] and this nomenclature will be used for the rest of this communication.

Prognostic favorable outcome of MFS depends on early diagnosis and aggressive but limb-sparing treatment [1]. The most important prognostic factor identified so far is the quality of surgical treatment at initial diagnosis [2]. Meanwhile, new prognostic factors are sought in order to specifically assess the importance of an adjuvant or palliative therapy.

Cell-to-cell and cell-to-matrix interactions are essential for normal cell growth and differentiation, though their precise function remains unclear. A variety of adhesion molecules participate in these interactions including the hyaluronic acid binding cell surface glycoprotein CD44. CD44 is expressed on all cell types and acts as a receptor for hyaluronate [12,13]. Therefore, CD44 may play a role in cellular differentiation/migration and cell-to-cell-contact. In aberrant gene expression within sarcomas, CD44 could play a role especially for invasion, such that may lead to lymph node or distant metastases and thus plays a role in prognosis.

CD44’s encoding gene is located on chromosome 11p3 and consists of at least 21 exons [14]. The CD44 molecule consists of three core epitopes encoded by ten exons with alternative mRNA splicing of the remaining exons generating multiple isoforms. The standard form of CD44 (CD44s) is expressed on almost all cells and is heavily glycosylated, while variant isoforms are expressed in a cell- and tissue-specific manner [15]. Post-translational modifications, such as glycosylation and alternative splicing, add even further to the diversity of the function of the special CD44 isoforms.

The standard form of CD44 as well as its variants is widely expressed in several tissue types but there are conflicting reports about the associations between CD44v and patients’ prognosis. High levels of CD44 and CD44v have been associated with poor outcomes in breast cancer, gastric cancer, colorectal carcinoma and head/neck cancer [3-6]. However, several studies have shown that CD44 expression is associated with a better outcome in cancers such as bladder tumors and other studies have suggested no direct association of CD44 with prognosis in cancers such as skin tumors [7,8].

Few studies have investigated CD44 expression by STS, especially in MFS. In an earlier immunohistochemical investigation, we determined that CD44s-positive STS cells significantly correlated with longer survival [9]. The aim of this study was to evaluate the isoforms of CD44 expression in adult STS and to determine whether this could provide prognostic information.

## Methods

This is a retrospective multicenter pilot study performed on MFS tissue obtained from the tumor banks of the Department of Surgery, Heinrich-Heine-University, Düsseldorf, and the Department of Plastic Surgery, Ruhr University, Bochum. Patient data were collected retrospectively starting at the time of specimen collection until the end of the study observation period. The study was approved by the local ethical commission board from the University of Dusseldorf. (Study No. 4487). All tumor specimens were reviewed by one experienced pathologist as well as a national sarcoma reference center to confirm the diagnosis of MFS.

Pretreatment evaluation included a complete history and physical examination, chest CT or chest radiograph, and computed tomographic or magnetic resonance imaging scans of the tumor site as well as routine ECG and pulmonary function tests, if needed. In all cases, treatment options were discussed in an interdisciplinary tumor conference. All patients were subsequently resected with a curative intention. In three patients, resection was performed despite distant metastases either because of the young age or the explicit wishes of the patient. Based on the tumor size and anatomic site, either a complete/compartment resection or wide local excision was performed. Tumor staging was performed according the UICC TNM-System (1997).

Frozen tumor samples were cut in 20-μm thick sections with a cryostat. Ten sections were immediately placed in an Eppendorf tube and total RNA was extracted by using the TRIzol reagent (Invitrogen, USA) according to the manufacturer’s instructions. RNA concentration was verified spectrophotometrically (BioPhotometer, Eppendorf, Germany) by using the OD260 method. Reverse transcription was performed in a volume of 20 μL by using random hexamer primer, 2 μg RNA and reverse transcriptase in 5× RT-buffer (all from Roche Diagnostics, Germany). Quantitative PCR with cDNA was performed using the iQ Sybr Green Supermix (BioRad). PCR conditions and primer sequences for CD44, CD44 isoforms, and the reference genes beta-actin and beta-microglobulin, were selected as previously described [10] with the exception that the annealing temperature for CD44v8 was changed to 58°C and that of CD44s to 54°C. For relative quantification and to normalize the amount of the CD44 product and the CD44 isoform products, we applied the delta-CT method using the expression of the reference genes beta-actin or beta-microglobulin, respectively. The expression rate of CD44 was correlated to the TNM category, resection status, grading, tumor size, and tumor free and overall survival.

### Statistical analysis

Data analysis was performed through grouping of the measured expression by separating patients into two equally sized groups at the median (high and low expression) for each isoform. Survival curves were determined using the Kaplan-Meier estimator and compared by the log-rank test with 95% confidence intervals based on Greenwood’s formula [16]. Prognostic risk factors of patient survival (TNM-category, tumor size, resection margin status, histology, grade, and patient age) were evaluated by univariate and multivariate analysis. The Cox proportional-hazards regression model was used to determine risk factors and hazard ratios [17]. Two-tailed values of *P* <0.05 were considered significant.

## Results

In total, we examined 34 adult MFS patients (19 males and 15 females, average age 62 years, median 63 years, ranged from 38 to 88 years). MFS was confirmed in all tumor specimens after surgical treatment. Eleven patients underwent operation for recurrent disease, while 23 patients were operated on the primary tumor. The majority of patients received adjuvant radiotherapy (n = 25), while no patient received adjuvant chemotherapy. All MFS tumors were graded G3. Complete histological evaluation of the tumor specimen revealed four T1 tumors (12%), while 30 patients had a tumor of >5 cm in diameter (T2, 88%). In all patients, regional lymph nodes were either clinically or histologically without metastases (100%). Three patients presented with synchronous distant metastases (11%), while staging procedures revealed M0 in 31 patients (89%). In all four patients with a subcutaneous tumor (12%), a wide resection resulted in four R0 resections. In the 30 patients with a subfascial MFS, four patients (12%) had a compartmental resection performed, resulting in R0. Twenty-six tumors were resected with a wide excision, achieving tumor-free margins in 23 patients (68%). In three patients (8%), an R1 resection with narrow margins was performed to preserve large nerves surrounded by the tumor, to drain a seroma by primary incisional biopsy during resection, or due to a patient denying primary amputation (Table 1).

**Table 1:** Characteristics of the patients

	**Patients (n)**	**Patients (%)**
pT1-category	4	12
pT2-category	30	88
cN0	34	100
cM0	31	89
cM1	3	11
R0	31	89
R1	3	11

The mean survival was 54 ± 6 months. The tumor recurred locally in one patient after resection (3%). Distant metastases developed in nine patients (26%) after a median of 19 months (range 4–48 months). At the end of the follow-up period, 24 patients (71%) were without evidence of disease; all patients that developed distant metastases (n = 19, 29%) died from their tumor within a median of 24 months.

**Figure 1:**
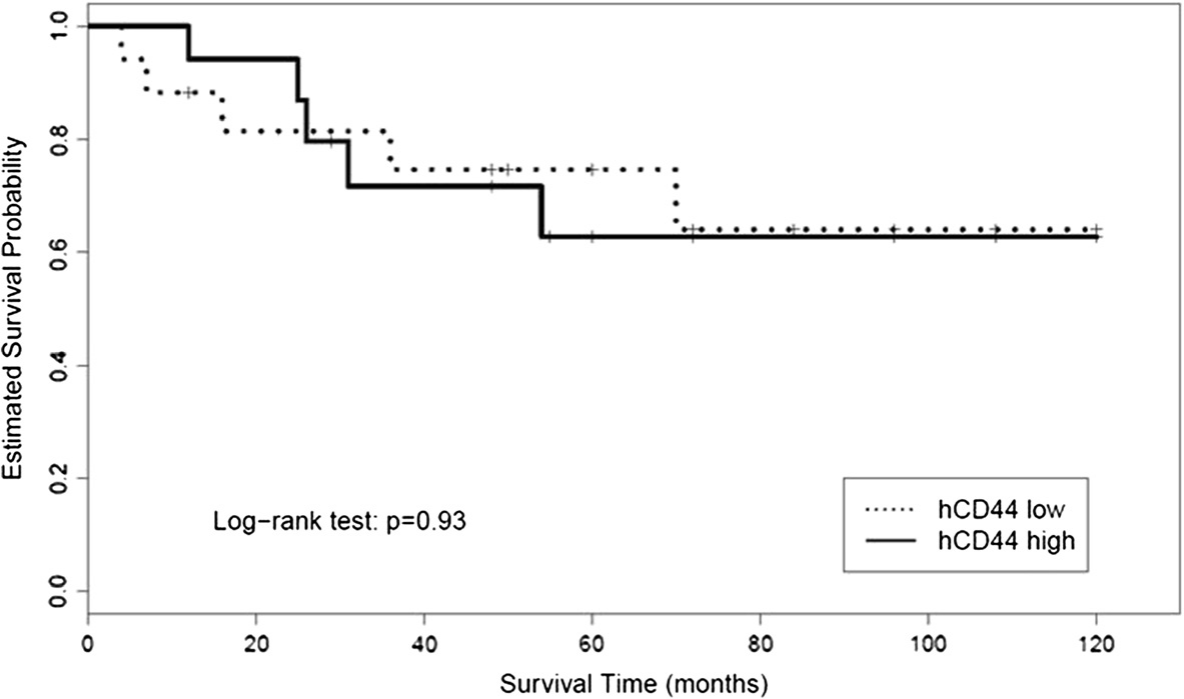
Kaplan-Meier estimates for hCD44.

**Figure 2:**
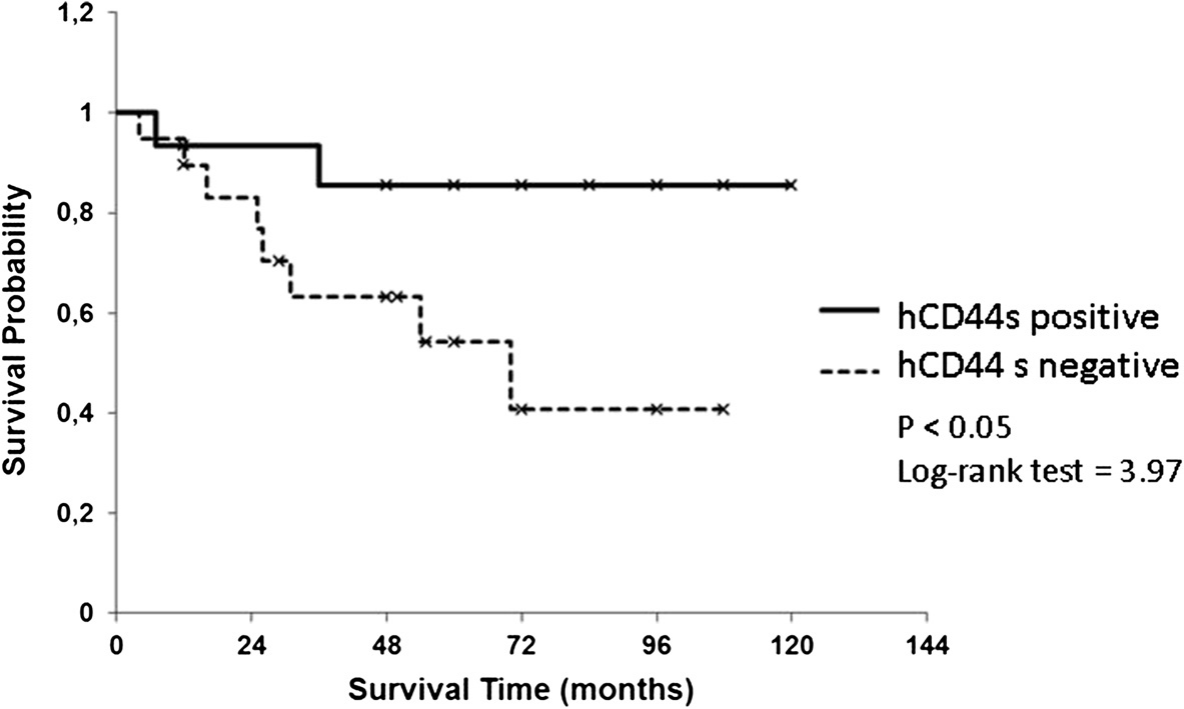
Kaplan-Meier estimates for hCD44s.

**Figure 3:**
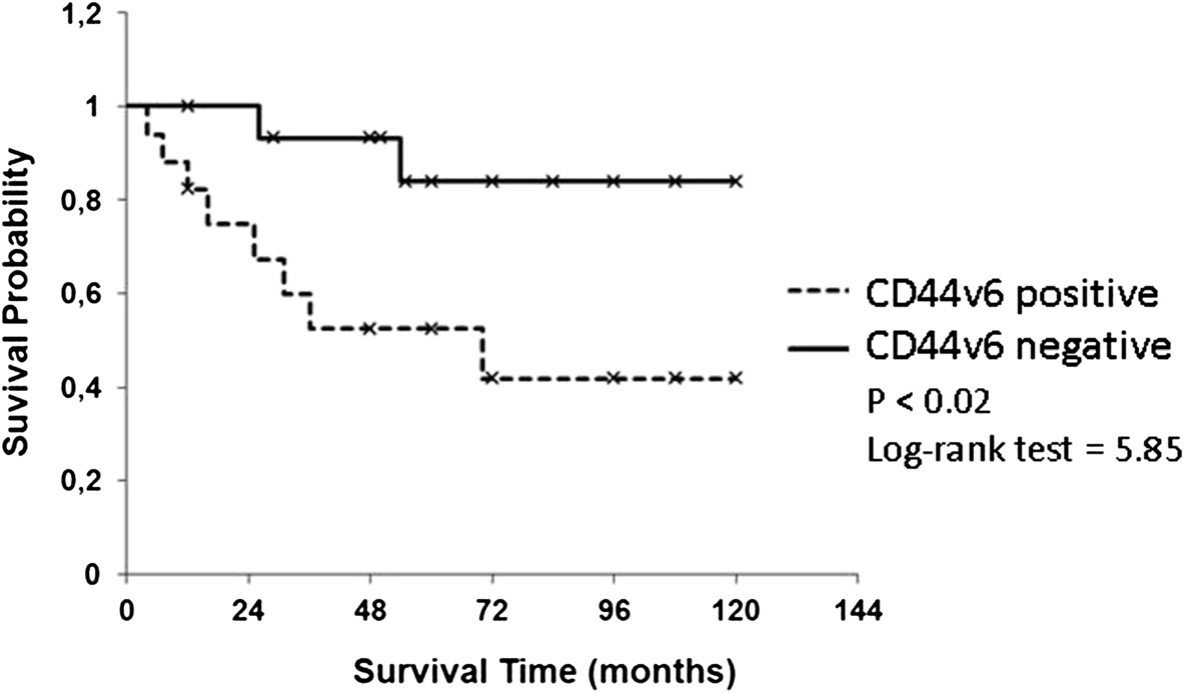
Kaplan-Meier estimates for hCD44v6.

**Figure 4:**
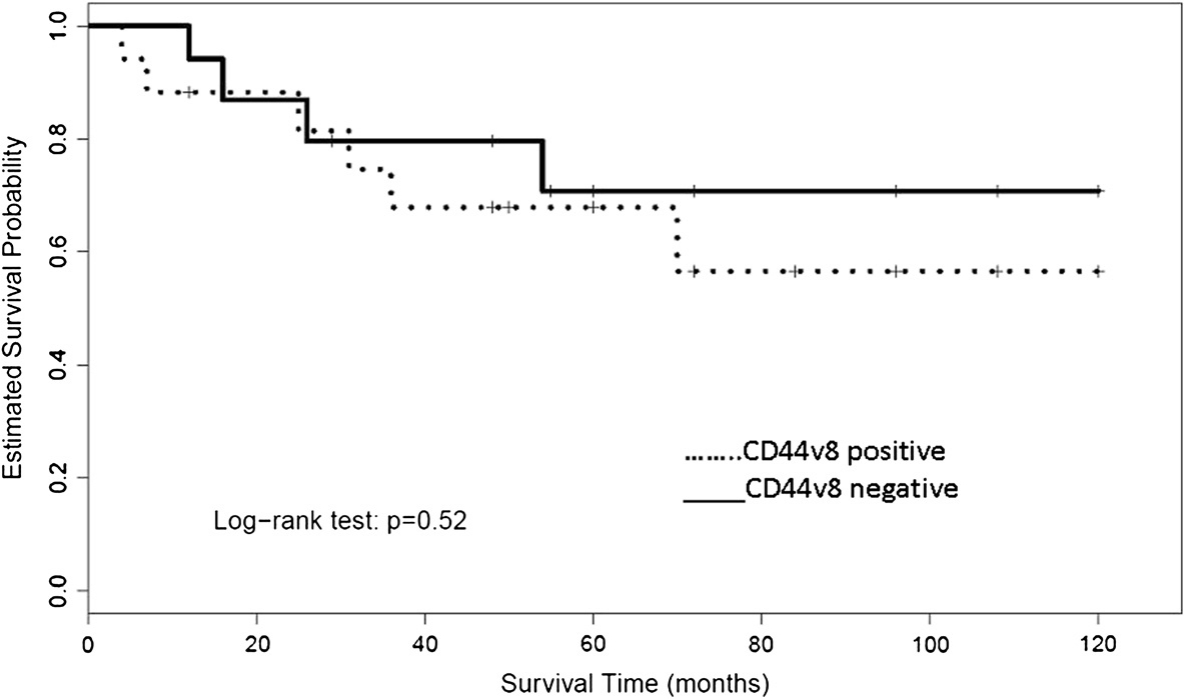
Kaplan-Meier estimates for hCD44v8.

The grouping into two groups by the analyzed variants of CD44 (hCD44, hCD44s, hCD44v6, and hCD44v8) showed a significant difference in tumor related survival only for CD44s and CD44v6 (*P* <0.05 and *P* <0.02, respectively). Visualization of the Kaplan-Meier estimates for the four isoforms of CD44 is shown in Figures 1, 2, 3, and 4. In patients with an increased expression of the hCD44s isoform, there was a significantly longer overall survival with a 5-year survival rate of >80% compared to 50% in patients with a loss of hCD44s. Furthermore, we noticed a survival benefit in patients with a loss of CD44v6 isoform, again with a 5-year survival of >80% compared to 50% in patients with a CD44v6 positive sarcoma. The survival curves begin to diverge as early as 6 months after resection.

CD44s and CD44v6 were independent predictive markers for the outcome of our patients when it was controlled for stage, tumor grade, age and margin status.

However, a significant correlation to the TNM-category, especially to metastases, the histology of the specimen, resection status and tumor size could not be found. This may be due to the low number of M1 and R1 cases with all specimens graded G3. Correlation between CD44 expression and tumor depth did not quite reach a significant value (*P* >0.5).

## Discussion

Qualitative and quantitative changes in expression of CD44 and its isoforms have been demonstrated for several tumor models revealing prognostic significance for neuroblastoma [18], breast cancer [13], squamous cell esophageal cancer [19], osteosarcoma [20], and gastric cancer [21]. Only few reports exist about survival and CD44s expression in STS [9] and rhabdomyosarcoma [15]. We could not find any report focusing on MFS sarcomas. A recent study examined CD44v expression in a variety of STS and noted that expression of CD44v6 is more frequently detected in high-grade than in low-grade tumors [22]. They described a correlation with metastases-free survival in these patients although overall patient number was small. Maula et al. observed similar results in a recently published observation of limb or superficial STS in Finland [23]. In an analysis of 62 patients with several STS entities, we detected a prognostic benefit if CD44s was highly expressed [9].

Here, we found significant differences between the CD44 high expression group and CD44 low expression group regarding overall survival. Though the role of CD44 in metastasis remains unclear, CD44 may be important during invasion of the target organs, perhaps by interaction of the molecules with special ligands (e.g., collagen, fibronectin, osteopontin) [24]. Changes in adhesion molecules that participate in cell-cell boundaries and cell-matrix interactions are a prerequisite for tumor invasion and dissemination. In STS, expression of cell-surface antigen CD44 has been associated with local recurrence [23] and, in particular, the isoform CD44v6 has been correlated with metastatic disease [22]. However, we could not confirm these results with our data. The measured CD44 isoforms did not correlate with metastases in our patients. Our results are in line with a study by Kahara et al. [22], where they found that increased CD44v6 at the protein level was associated with a poor clinical outcome. Nilbert et al. found no differences in the expression of CD44 in MFS primary tumors, local recurrences or metastases, nor could they find any correlation between CD44 and prognosis [25]. However, it should be noted that the CD44v isoform was not analyzed, which could possibly explain the lack of prognostic importance.

In our study, the strong correlation between CD44 expression and actuarial survival revealed CD44 as a strong prognostic marker. CD44 expression is not the only independent prognosticator for STS; in fact, previous studies have shown that the resection margin and tumor size represent independent prognostic indicators [25]. Tumor depth almost reached significance in our study but other well-described factors, such as tumor grade, did not. This may be due to the relative homogeneity of our patient cohort with all G3 tumors and few M1 at diagnosis or R1 resections. However, the relative homogeneity of the cohort is also strength to the study as it may help to inform decisions about this relatively common cohort among MFS patients. Future studies with larger and more diverse cohorts are needed to define the precise role of this cell adhesion molecule in invasion and metastases of MFS. Nevertheless, the association between CD44s expression and progression of MFS is important, as it suggests that CD44s may play a protective role in tumor progression.

The overexpression of CD44v6 in some tumor types has been correlated to metastatic potential. The different CD44v proteins originate in alternative splicing of variant exons at the copying stage and are expressed in several cancer types. CD44v6 overexpression was found in metastatic breast cancer [26]. Recently, overexpression of CD44v6 was described in squamous cell cancer with a significant correlation between abnormally high expression of CD44v6 and infiltration in surrounding tissue as well as metastatic disease [27]. CD44v6 was only expressed in metastatic cell strains, but not in non-metastatic cell lines [11]. The non-metastatic cell lines gained metastatic potential after transfection with CD44v6.

While CD44s is a candidate tumor suppressor in MFS, CD44v6 may function as an oncogene. This is consistent with prostate cancer, in which CD44s is a tumor suppressor but certain CD44 variants are oncogenes and promote growth [28,29]. Since MFS is a heterogeneous group of tumors nowadays often classified as sarcoma not otherwise specified (NOS), several tumor entities may be summarized under this term. Further classification of MFS-NOS does not exist at present but clinical experience has shown that there are patients with MFS-NOS with an extremely poor prognosis, while others can be cured through surgery alone. A positive impact of chemotherapy on patient survival has been seen in osteosarcoma with CD44 expression [20]. Therefore, a prospective cohort study design should be performed to analyze if a treatment escalation can improve the clinical outcome of these patients. Further evaluation of CD44v6 expression as an important prognostic and predictive biomarker is needed before clinical use.

## Conclusions

Our study suggests that low expression of CD44s as well as high expression of CD44v6 correlates with poor outcome. Although the cohort was relatively homogenous, these correlations were independent of other survival predictors; thus, further studies are needed to determine if they may be clinically useful.

## Abbreviations

MFS: Malignant fibrous sarcomas; NOS: Not otherwise specified; STS: Soft tissue sarcomas.

## Competing interests

All authors have no disclosure of any financial and personal relationships with other people or organizations that could inappropriately influence (bias) their work.

## Authors’ contributions

CP examined the tissue material carried out the molecular biological examinations. MP and ML collected the tissue material. MP, CM conceived the study, participated in the design of the study and prepared the manuscript. MP performed the statistical analysis. All other authors helped to draft the manuscript. All authors read and approved the final manuscript.
